# Mechanism of Cu(II) Detoxification by Ergothioneine: Reduction and Formation of Stable Cu(I) Complexes

**DOI:** 10.3390/antiox15091064

**Published:** 2026-08-25

**Authors:** Yuri P. Tsentalovich, Nataliya A. Osik, Maxim V. Fomenko, Nikita A. Dmitriev, Vadim V. Yanshole

**Affiliations:** Laboratory of Proteomics and Metabolomics, International Tomography Center SB RAS, Institutskaya Street 3a, 630090 Novosibirsk, Russia; n.osik@tomo.nsc.ru (N.A.O.); m.fomenko@tomo.nsc.ru (M.V.F.); n.dmitriev1@g.nsu.ru (N.A.D.); vadim.yanshole@tomo.nsc.ru (V.V.Y.)

**Keywords:** ergothioneine, copper, ergothioneine disulfide, hercynine, oxidation kinetics, complexing, optical spectroscopy, NMR, LC-MS

## Abstract

Ergothioneine (ESH) is one of the most abundant antioxidants in the human body, but its biological functions remain unclear. In this work, we elucidated the detailed mechanism of the deactivation of divalent copper ions Cu(II) by ESH using optical spectroscopy, NMR, and LC-MS. We found that the reduction of Cu(II) ions to Cu(I) by ESH occurs in a bimolecular reaction of Cu^II^(ES^−^)_2_ complexes, leading to the formation of the disulfide ESSE. Cu(I) ions remain bound in stable complexes with ESH, preventing their reactions with molecular oxygen. Over longer timescales, ESSE decomposes, regenerating reduced ESH and forming the final reaction product, hercynine (EH). The conversion of one ESH molecule to EH causes the reduction of four Cu(II) ions. We determined the rate constants of the bimolecular reaction between Cu^II^(ES^−^)_2_ complexes to be k_3_ = (8 ± 4) × 10^4^ M^−1^s^−1^ and of ESSE hydrolysis to be k_4_ = (1.7 ± 0.4) × 10^−5^ s^−1^ and also estimated the formation constant K_12_ ≈ 1.2 × 10^17^ M^−2^ for the Cu^II^(ES^−^)_2_ complex. The high efficiency of Cu(II) reduction and the chelation of the resulting Cu(I) ions in a stable complex supports the hypothesis that protecting cells from oxidation by metal ions is likely one of the main functions of ESH.

## 1. Introduction

**Figure 1:**
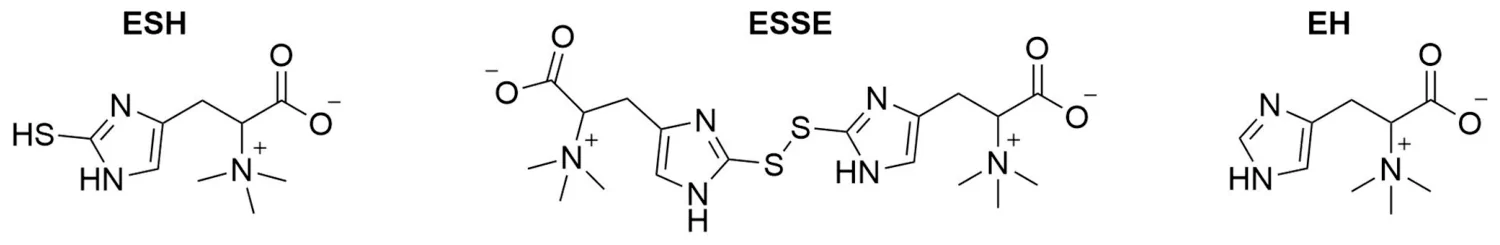
Chemical structures of ergothioneine (ESH), ergothioneine disulfide (ESSE) and hercynine (EH).

Redox reactions play a vital role in almost all biological processes. The generation of reactive oxygen and nitrogen species (ROS and RNS), radicals, and other highly reactive species occurs in all living cells. Protection of cell components from excess active species is largely provided by antioxidants—compounds able to prevent or delay oxidative damage to tissues. The most abundant antioxidants in the human body are glutathione (GSH), uric acid, and vitamins C (ascorbate), A (retinol), and E (α-tocopherol), which efficiently scavenge ROS, RNS and radicals; quench reactive photoexcited triplet states; and inhibit lipid peroxidation [1,2,3,4]. GSH, a tripeptide consisting of cysteine, glutamate, and glycine, is produced by intracellular synthesis; uric acid is the product of purine metabolism; and vitamins appear in the human body only from the diet. One more very abundant antioxidant in the human body, ergothioneine (ESH, 2-Thiol-L-histidine-betaine, Figure 1), is also a diet-derived metabolite: the synthesis of ESH occurs only in fungi and some bacteria [5]. The ESH level in mushrooms reaches 0.2 g per 100 g dry weight [5,6]. Therefore, mushrooms are the most common direct source of ESH for humans [6].

Recently, scientific interest in the properties of ESH has sharply increased: in the last several years, hundreds of original papers and dozens of reviews have been published that are directly related to ESH. A significant portion of these publications is devoted to the antioxidant properties of ESH, its content in various food products, its distribution in animal tissues, as well as its biological role and potential therapeutic applications [5,6,7,8,9,10]. However, the exact biological functions and chemical mechanisms underlying the biological role of ESH remain unclear. On the one hand, the accumulation of large amounts of ESH in tissues most exposed to oxidative stress (up to several mM in erythrocytes, liver, and the eye lens [5,7,11,12,13,14,15]) indicates the importance of this compound for antioxidant protection. ESH uptake from the gastrointestinal tract and accumulation in tissues occurs with the help of the ESH-specific transporter OCTN1 [16,17]. OCTN1 is highly expressed in different human and animal tissues including the kidney, lung, trachea, small intestine, cord blood, fetal liver and bone marrow [18], and the very existence of this protein in animals underscores the importance of ESH for normal cell functioning. ESH has been shown to have therapeutic effects in a number of diseases, including lung injury and inflammation, fatty liver disease, skin damage, and COVID-19 [19,20,21]. On the other hand, no diseases caused by ESH deficiency have been reported so far. Mice with inactivated OCTN1 gene [6] have very low levels of ESH in all tissues but do not show phenotypic changes. A comparison of the antioxidant properties of ESH with those of other antioxidants shows that ESH has relatively low reactivity with ROS [6,11,22]. ESH scavenges highly reactive hydroxyl radical (^•^OH) with a near-diffusion-controlled rate constant [23,24] and effectively neutralizes oxidants such as hypochlorous acid (HOCl) [24] and peroxynitrite (ONOO^−^) [25,26]. Compared to other antioxidants such as glutathione, vitamin C, or vitamin E, ergothioneine possesses a higher redox potential, indicating lower reactivity in reactions with oxidizing agents [22]. On the other hand, a study [26] demonstrated that ESH scavenges ^•^OH, ^•^OOH, and ONOO^−^ more effectively than GSH, ascorbate, and trolox (a vitamin E analog). Moderate reactivity of ergothioneine toward the superoxide anion radical (O_2_^•−^) and hydrogen peroxide (H_2_O_2_) has been reported in studies [24,27,28]. Some researchers [29,30,31] suggest that ESH is largely uninvolved in primary cytoprotection and only comes into play when other antioxidant defense mechanisms are exhausted.

However, there is one thing that ESH does perhaps better than other antioxidants, namely, chelating metal ions, thus preventing them from participating in oxidation reactions [11,32,33,34]. It has been shown that ESH prevents copper-induced oxidative damage to DNA, proteins and sugars [24,35] by forming a redox-inactive ergothioneine–copper complex. ESH has been reported to form chemically stable complexes with monovalent Cu(I) [33,35]. Many publications also suggest that ESH forms stable complexes with divalent Cu(II) ions [7,11,32,33,36], but some authors point to the possibility of a redox reaction leading to the oxidation of ESH and the reduction of Cu(II) to Cu(I) [33,37]. Thus, the existence of stable complexes with divalent Cu(II) is under debate, and the mechanism of the ESH interaction with Cu(II) ions remains largely unexplored. It is worth noting that the above-mentioned publications mainly focused on the ultimate biological action and role of ESH, while the mechanistic aspects of the ESH reaction with Cu(II) remain less studied.

In this work, we performed a comprehensive study of the reactions between ESH and Cu(II) using a combination of optical spectroscopy, NMR spectroscopy, and LC-MS. The aim of this work is to establish a detailed mechanism of ESH oxidation by Cu(II) ions, determine the main reaction products, measure the rate constants of the elementary stages of the reaction, and evaluate the formation constants for complexes of copper ions and ESH.

## 2. Materials and Methods

### 2.1. Chemicals and Solvents

L-Ergothioneine (ESH, CAS: 497-30-3) from Aladdin Scientific (Riverside, CA, USA), copper(II) chloride (CuCl_2_, CAS: 7447-39-4) from Reachem (Moscow, Russia), copper(I) chloride (CuCl, 7758-89-6), 2-(N-morpholino)ethanesulfonic acid (MES, CAS: 145224-94-8) hydrate, sodium hydroxide (NaOH, CAS: 1310-73-2), formic acid (CAS: 64-18-6) from Sigma–Aldrich (St. Louis, MO, USA), mono- and disodium phosphate (NaH_2_PO_4_, CAS: 13472-35-0, and Na_2_HPO_4_, CAS: 7558-79-4, respectively) from Applichem (Darmstadt, Germany), sodium 2,2-dimethyl-2-silapentane-5-sulfonate (DSS, CAS: 2039-96-5) from Cambridge Isotope Laboratories, Inc. (Andover, MA, USA), D_2_O 99.92% (CAS: 7789-20-0) from Astrachem (St. Petersburg, Russia) and acetonitrile “0” grade (CAS: 75-05-8) from Cryochrom (St. Petersburg, Russia) were used as received. Deionized water was obtained using an Aqualab AL-1 Plus water treatment system (Moscow, Russia) to a quality of 18.2 MOhm × cm.

### 2.2. Buffer Preparation

Phosphate buffer solutions were prepared by dissolving Na_2_HPO_4_ and NaH_2_PO_4_ in D_2_O or deionized H_2_O. The concentrations of the buffer components were determined by the required total phosphate concentration and the pH of the buffer. MES buffer solutions were prepared by dissolving MES hydrate in D_2_O or deionized H_2_O and adding small volumes of 1 M NaOH to obtain the required pH value of the final solution. The pH of buffer solutions was measured by a FiveEasy Plus FP20 pH-meter equipped with an InLab Semi-Micro pH sensor from Mettler Toledo (Columbus, OH, USA). DSS at a concentration of 20 µM was added to the phosphate and MES buffer solutions in D_2_O as an internal chemical standard for further NMR measurements.

### 2.3. Optical Measurements

Steady-state and time-resolved UV–Visible absorption measurements were performed using an Agilent 8453 spectrophotometer (La Jolla, CA, USA). All optical measurements were carried out in the 190–800 nm spectral range for 3 mL solutions of the reaction mixture placed in a 1 × 1 cm^2^ quartz cell. During the time-resolved measurements, full optical absorption spectra were recorded at certain time intervals (from 0.5 s to 3 s depending on the reaction rate), while the kinetics of the chemical processes was monitored at 257 nm and 317 nm. The temperature in the cell was maintained at 25 °C with an Agilent 89090A temperature control unit (La Jolla, CA, USA), and the solution was constantly stirred with a magnetic stirrer.

### 2.4. NMR Measurements

For steady-state and time-resolved ^1^H NMR measurements, 600 µL of freshly prepared mixture of ESH and CuCl_2_ in 50 mM phosphate or 5 mM MES buffers in D_2_O was placed in a 5 mm NMR tube. The ^1^H NMR spectra were recorded using an AVANCE III HD 700 MHz NMR spectrometer from Bruker BioSpin (Rheinstetten, Germany) equipped with a 16.44 Tesla Ascend cryomagnet at the Center of Collective Use “Mass spectrometric investigations” SB RAS.

1D NMR spectra were recorded using a standard Bruker “zgpr” pulse sequence with low-power presaturation of the water signal and a 70-degree detection pulse with the acquisition time (AQ) and relaxation delay (d1) set to 3.5 s and 10 s, respectively. The number of scans for each spectrum was set to 64, which resulted in a total acquisition time of 14 min 53 s. The NMR tube temperature was set to 25 °C, and the probe spinning rate was set to 20 Hz. We performed phase and baseline corrections as well as signal integration using MestReNova v.12 software from Mestrelab Research S.L. (A Coruna, Spain). The concentration of all components in the reaction mixture was calculated by integrating the corresponding NMR signals relative to the signal of the internal standard DSS.

To conduct the kinetic NMR experiment, we sequentially recorded 85 NMR spectra using the program described above. Thus, we obtained time-resolved kinetics of all components of the reaction mixture over 21 h with a 15 min time interval.

### 2.5. Liquid Chromatography with Optical and Mass Spectrometric Detection (LC-OD-MS)

To identify the reaction products, we carried out LC separation of the reaction mixture with combined optical and mass spectrometric detection as described in our previous paper [38]. The measurements were performed using an UltiMate 3000RS chromatograph from Dionex (Germering, Germany) equipped with a flow cell diode array UV-Vis detector (DAD) with the spectral registration in the range from 190 nm to 800 nm that was subsequently connected to an ESI-Q-TOF high-resolution hybrid mass spectrometer maXis 4G from Bruker Daltonics (Bremen, Germany). LC separation was carried out using reversed-phase chromatography on a Zorbax Eclipse XDB C18 column (4.6 × 250 mm, 5 μm, Agilent Technologies, La Jolla, CA, USA). Solvent A consisted of 0.1% formic acid solution in H_2_O, and solvent B consisted of 0.1% formic acid solution in acetonitrile. The following gradient (solvent B) was used: 0% (0–5 min), 0 to 40% (5–15 min), 40 to 95% (15–17 min), 95% (17–21 min), 95 to 0% (21–23 min), and 0% (23–29 min). The flow rate was 1 mL/min, the injection volume was 5 μL, and the autosampler and column temperatures were 25 °C and 40 °C, respectively. Mass spectra were recorded in positive mode in the mass range from 50 to 1000 *m*/*z* with a 3 Hz scan rate. Each LC-MS chromatogram contained a calibration segment at the beginning with sodium formate clusters used as a calibrant. The typical MS resolution was approximately 50,000, and the accuracy was <1.5 ppm. We also performed LC-MS/MS fragmentation experiments for the reaction mixture 24 h after mixing. We used two fixed collision induced dissociation (CID) energies of 10 eV and 20 eV and performed consecutive runs in AutoMS/MS acquisition mode with a precursor ion list.

### 2.6. LC Fraction Collection

Additional LC-MS runs were performed to gather and accumulate reaction products. We increased the injection volume to 70 µL and selected fixed retention time (RT) windows for products to collect them as separate LC fractions. After passing through a flow optical cell, a home-made flow splitter (1:10) directed the smaller flow to the MS system, while the majority of the flow was collected into separate vials during 7–8 consecutive LC runs. Combined fractions were vacuum dried, dissolved in deuterated phosphate buffer (pH 7.0) and subjected to NMR analysis.

### 2.7. Theoretical Calculations

Numerical calculations of processes occurring in the solution after mixing ESH with Cu(II) ions were conducted using a program code written in Matlab version 8.5 R2015a (The MathWorks, Inc., Natick, MA, USA) employing the Matlab function “roots” to solve polynomial equations and “fminsearch” to fit the experimental data by the least squares method. The errors in the reported values of the rate constants were determined on the basis of the fitting confidence intervals.

## 3. Results

### 3.1. Characterization of Reaction Products Using Spectrophotometry, LC-OD-MS and NMR

**Figure 2:**
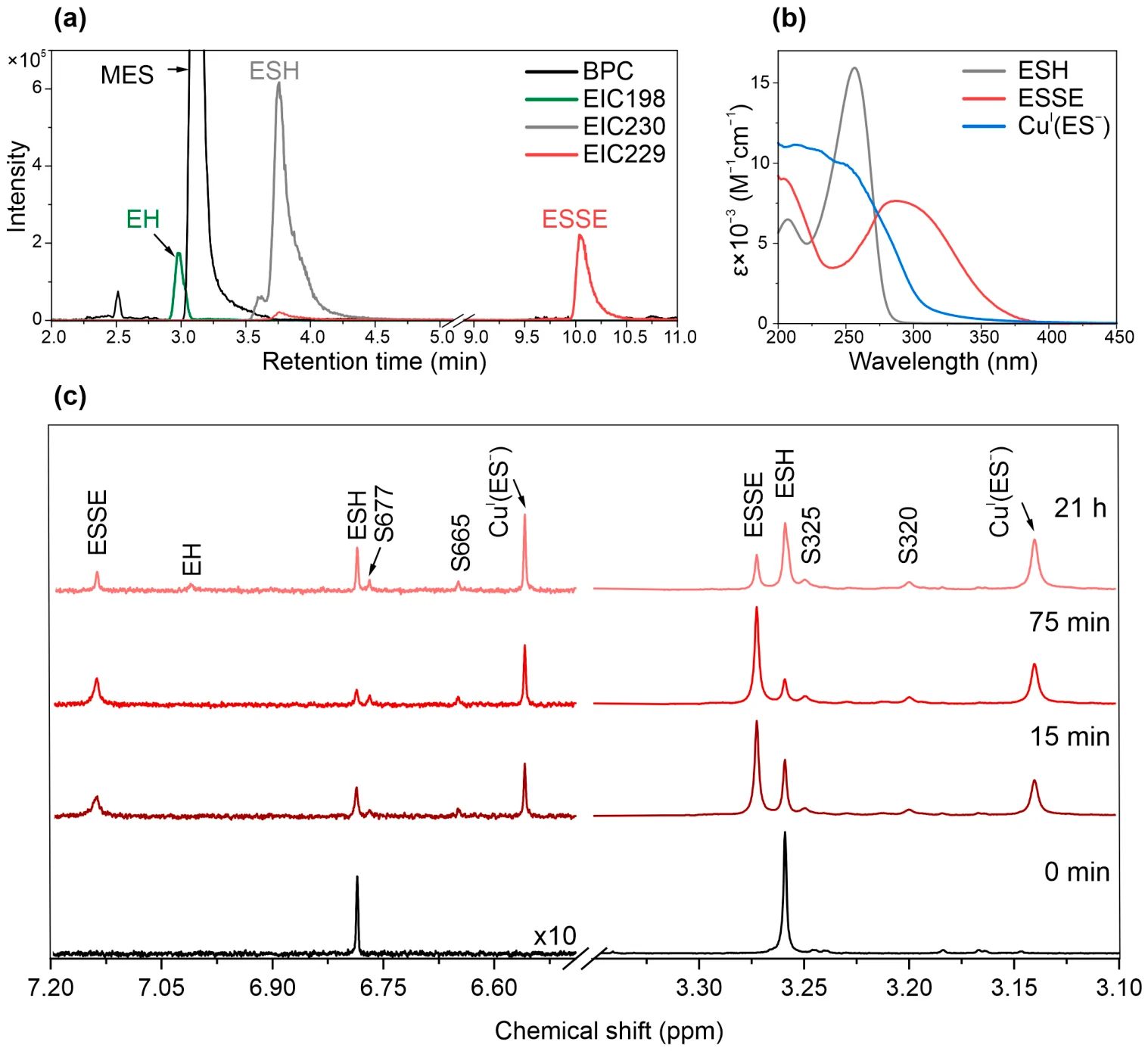
(**a**) BPC (black line) and EICs for *m*/*z* 198.1237 (green line), 230.0958 (gray line), and 229.0879 (red line) obtained with LC-MS 24 h after mixing CuCl_2_ and ESH in MES buffer. (**b**) UV-Vis spectra of ESH, ESSE and Cu^I^(ES^−^). (**c**) Aromatic and aliphatic regions of ^1^H NMR spectra of ESH in deuterated phosphate buffer before (lower spectrum) and 15 min, 75 min, and 21 h after adding CuCl_2_.

We carried out chromatographic separation of the reaction mixture with simultaneous UV-Vis and mass spectrometric detection (LC-OD-MS). To determine the intermediate and final reaction products formed upon ESH and Cu(II) mixing, 2 × 10^−4^ M CuCl_2_ and 4 × 10^−4^ M ESH were mixed in 1 mL of 5 mM MES buffer (pH 6.0), and then 5 µL of the mixture was immediately injected into the column. The injection was repeated 0.5 h, 1 h, 2 h, and 24 h after mixing. The base peak chromatogram (BPC) of the MS signal as well as the extracted ion chromatograms (EICs) of the reaction products obtained during the separation are shown in Figure 2a, while the MS/MS spectra of the eluted compounds are given in the SI (Figures S1–S3). The major components detected in the mixture were ESH (RT = 3.80 min, *m*/*z*_obs_ = 230.0958, *m*/*z*_calc_ = 230.0958 for C_9_H_16_N_3_O_2_S^+^; λ_max_ = 258 nm), ergothioneine disulfide ESSE (RT = 10.10 min, doubly charged *m*/*z*_obs_ = 229.0879, *m*/*z*_calc_ = 229.0879 for C_18_H_30_N_6_O_4_S_2_^2+^, and singly charged *m*/*z*_obs_ = 457.1680, *m*/*z*_calc_ = 457.1686 for C_18_H_29_N_6_O_4_S_2_^+^; λ_max_ = 300 nm), and hercynine EH (RT = 3.01 min, *m*/*z*_obs_ = 198.1236, *m*/*z*_calc_ = 198.1237 for C_9_H_16_N_3_O_2_^+^; λ_max_ = 210 nm). The chemical structures of ESH, ESSE and EH are shown in Figure 1. We found that the concentration of ESSE first increased and then decreased with time, the concentration of EH increased monotonously, and the concentration of ESH decreased during first 30 min and then increased. The same results were obtained in the LC-MS experiment with the ESH and Cu(II) mixture in phosphate buffer (50 mM, pH 7.0).

Chromatographic fractions containing ESH, ESSE, and EH were collected, vacuum dried, re-dissolved in deuterated phosphate buffer (pH 7.0), and subjected to NMR measurements. All three compounds have distinct singlet NMR signals corresponding to (CH_3_)_3_-groups and aromatic CH protons: ESH—3.259 ppm (9 protons) and 6.785 ppm (1 proton); ESSE—3.272 ppm (18 protons) and 7.138 ppm (2 protons); EH—3.261 ppm (9 protons), 7.010 (1 proton), and 7.760 ppm (1 proton).

Next, we mixed 83 µM CuCl_2_ and 165 µM ESH directly in an NMR tube in deuterated phosphate buffer (pH 7.0), placed the sample into the NMR probehead, and acquired NMR spectra 15 min, 75 min, and 21 h after mixing (Figure 2c). In addition to the signals from ESH, ESSE, and EH, we observed additional NMR singlet signals in the aliphatic (3.140 ppm) and aromatic (6.558 ppm) regions with a ratio of 9:1. It is well known that copper and ESH in aqueous solutions form coordination complexes [33,39]. Since complexes with Cu(II) are paramagnetic and cannot be detected in NMR spectra, we attributed these signals to the coordination complex of Cu(I) with ESH formed in the reaction between ESH and Cu(II). To verify this attribution, we added an excess of CuCl suspension in D_2_O to an argon-bubbled 1 mM ESH solution in deuterated phosphate buffer and acquired NMR spectra 15 min, 1 h, 4 h, and 24 h after mixing. We found that within 24 h, the ESH concentration decreased to 0.4 mM, and the signals from the complex of Cu(I) with ESH (3.140 ppm and 6.558 ppm) appeared immediately after mixing. The concentration of the complex increased during the experiment, reaching 0.5 mM after 24 h. The obtained identification of NMR signals was later used for NMR-based kinetic measurements (Section 3.2).

We should note that the signals from the complexes of ESH and copper were not detected in the LC-MS experiment. Apparently, during chromatographic separation, a strong dilution of the sample with the acidic mobile phase occurs, the complexes dissociate in the column volume, and thus, we detected only the signal from the free ESH. The long tail observed after the main ESH peak (Figure S7) potentially indicates the release of ESH from the complex. We also tried to detect signals from the complexes using a direct injection of the reaction mixture to the ESI source, but we were unable to distinguish them from copper adduct ions of ESH formed during electrospray ionization.

The optical properties of the reaction products were determined using spectrophotometry in combination with NMR spectroscopy. NMR was used to measure the actual concentrations of substances in the solution. The electronic absorption spectrum of ESH (Figure 2b) was obtained with the use of 0.5 mM solution of ESH in phosphate buffer (pH 7.0). ESSE solution was prepared by collecting the ESSE fractions during the LC separation of the reaction mixture, drying it and redissolving in deuterated phosphate buffer (pH 7.0). Then, the NMR spectrum was obtained to determine the ESSE concentration in the solution using DSS as an internal standard, and the UV-Vis spectrum (Figure 2b) was measured with the use of a spectrophotometer. The obtained spectrum was corrected by subtracting the absorption of residual ESH, the concentration of which was quantified in the solution from the same NMR spectrum. To obtain the electronic spectrum of the complex of Cu(I) with ESH, we mixed the suspension of CuCl in D_2_O with a 1 mM ESH solution in deuterated phosphate buffer (pH 7.0) with an excess of CuCl and waited for 3 days for most of the ESH to be converted to the complex. We then filtered the solution and recorded the NMR and UV-Vis spectra of the solution, calculated the concentrations of the complex of Cu(I) with ESH and ESH by the NMR spectra, and corrected the obtained electronic spectrum of the complex of Cu(I) with ESH by subtracting the contribution of residual ESH.

### 3.2. Kinetic Optical Measurements

#### 3.2.1. Transient Absorption Spectra and Kinetics

**Figure 3:**
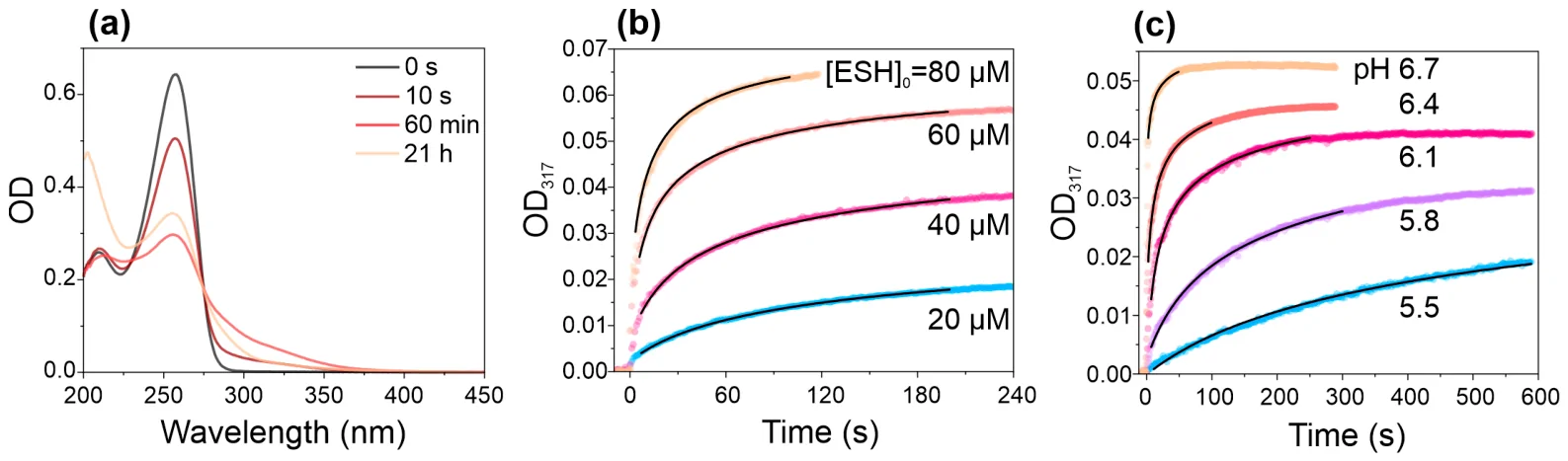
(**a**) Electronic absorption spectra detected before mixing and 10 s, 60 min, and 21 h after mixing 2 × 10^−5^ M CuCl_2_ and 4 × 10^−5^ M ESH in MES buffer (pH 6.0). (**b**) Kinetics of optical absorption monitored at 317 nm after mixing CuCl_2_ and ESH in MES buffer (pH 6.0). Concentration of CuCl_2_ is 2 × 10^−5^ M, and concentrations of ESH are indicated on the plot. (**c**) Kinetics of optical absorption monitored at 317 nm after mixing 2 × 10^−5^ M CuCl_2_ and 4 × 10^−5^ M ESH in MES buffer. pH values of solutions are indicated on the plot. Black lines in panels (**b**,**c**) show the simulations of ESSE formation in kinetic models (Section 3.4) according to the reaction scheme (1, 4–7) and the parameters: ε(ESSE) = 6 × 10^3^ M^−1^cm^−1^; K_12_ = 1.2 × 10^17^ M^−2^; R = 1; k_4_ = 1.7 × 10^−5^ s^−1^; k_3_ was the fitting parameter and varied from 5.5 × 10^4^ M^−1^s^−1^ to 7.8 × 10^4^ M^−1^s^−1^ (panel (**b**)) and from 7.2 × 10^4^ M^−1^s^−1^ to 1.3 × 10^5^ M^−1^s^−1^ (panel (**c**)).

Figure 3a demonstrates the electronic spectra, observed at different time intervals after mixing 2 × 10^−5^ M CuCl_2_ and 4 × 10^−5^ M ESH in MES buffer (5 mM, pH 6.0). Within the first hour after mixing, we observed the decay of the absorption at 257 nm, while the absorption in the 290–370 nm region with a maximum near 300–320 nm increased. Since the rates of processes observed at 257 nm and 300–320 nm are similar, one can assume that the signal decay monitored at 257 nm corresponds mainly to the consumption of ESH, and the signal growth at 300–320 nm shows the formation of a product of ESH decomposition. According to the spectral properties of the reaction products (Section 3.1), we attributed this product to ergothioneine disulfide ESSE. At a longer timescale, the signal at 300–320 nm passes its maximum and then decreases, indicating the slow decomposition of ESSE. Similar transient absorption spectra were obtained with the use of phosphate buffer (50 mM, pH 7.0), but the rate of the spectral changes was noticeably lower.

We checked the influence of oxygen on the kinetics of the reaction of Cu(II) and ESH in aqueous solution. We found that the kinetic curves obtained under air or O_2_ bubbling were the same as those obtained under argon bubbling (10 min prior to and during the measurements). Therefore, we concluded that the effect of molecular oxygen on the reaction is insignificant and conducted all further measurements under air.

It should be noted that several compounds contribute to the optical absorption at 257 nm, including the starting compounds, intermediate and final reaction products. This complicates the interpretation of the obtained kinetic curves at this wavelength. At the same time, at 317 nm, the absorption of ESSE is much stronger than that of all other compounds, and one can assume that the kinetic curves recorded at 317 nm almost precisely reflect the kinetics of ESSE formation and decay. Figure 2b demonstrates that the absorption coefficient of ESSE at 317 nm ε_ESSE_ = 6.0 × 10^3^ M^−1^cm^−1^ is much higher than that for ESH (below 10 M^−1^cm^−1^) and Cu^I^(ES^−^) complex (7.8 × 10^2^ M^−1^cm^−1^). For this reason, we performed all subsequent data treatment for traces recorded at 317 nm.

The rate of chemical transformations observed upon mixing CuCl_2_ and ESH in solution strongly depends on the initial concentrations of the reactants. As an example, Figure 3b shows the initial parts of the kinetic curves observed at 317 nm for the same initial concentration of CuCl_2_ (2 × 10^−5^ M) and concentration of ESH varying from 2 × 10^−5^ M to 8 × 10^−5^ M in MES buffer (5 mM, pH 6.0). With increasing ESH concentration in the solution, both the signal decay rate at 257 nm and the signal growth rate at 317 nm increase. At ESH concentrations above 10^−4^ M, the absorbance changes become too rapid for reliable measurements. A similar acceleration of ESH oxidation and ESSE formation is observed with an increase in the CuCl_2_ concentration at a constant ESH concentration (Figure S5).

In general, the oxidation of ESH by Cu(II) ions yielding disulfide ESSE can occur by two mechanisms—direct oxidation involving radical formation (reactions (2–3)) or through the coordination of Cu(II) ions with ESH and the stepwise formation of stable Cu^II^(ES^−^)_2_ complexes followed by the bimolecular reaction of two Cu^II^(ES^−^)_2_ complexes (reactions (4–6)):

ESH ⇄ ES^−^ + H^+^, → K_a_, (1)

Cu(II) + ES^−^ → Cu(I) + ES^•^, (2)

2ES^•^ → ESSE, (3)

or

(4) $$\mathrm{Cu}(\mathrm{II})+{\mathrm{ES}}^{-}⇄\mathrm{C1},\to K_{1},$$

(5) $$C1+{\mathrm{ES}}^{-}⇄\mathrm{C2},\to K_{2},$$

2C2 → ESSE + 2C3, → k_3_, (6)

where C1, C2 and C3 are (Cu^II^(ES^−^))^+^, Cu^II^(ES^−^)_2_, and Cu^I^(ES^−^) complexes, respectively; K_a_, K_1_, and K_2_ are the equilibrium constants of the corresponding reactions; and k_3_ is the rate constant of the bimolecular reaction (6).

We can assume that reactions (3), (4) and (5) are instantaneous on a second time scale, while the rate-determining steps are reaction (2) or reaction (6).

On a longer timescale, ESSE undergoes hydrolysis with the formation of the final reaction products, ESH, EH, and H_2_SO_3_ [27,28].

ESSE + 1.5H_2_O → 1.5ESH + 0.5EH +0.5H_2_SO_3_, → k_4_, (7)

The analysis of the kinetic curves obtained for different ESH concentrations (Figure 3b) indicates that the major mechanism of ESH oxidation is the bimolecular reaction of Cu^II^(ES^−^)_2_ complexes (reactions (4–6)). If direct oxidation of ESH (reactions (2–3)) were to occur, then with a large excess of ESH, the concentrations of ESH and ES^−^ may be considered constant, and reaction (2) would be expected to be a pseudo-first-order reaction with an observed rate constant proportional to the initial concentration of ESH. In the case of reactions (4–6), the second-order reactions are expected to occur for all ESH concentrations. Analysis of the initial parts of the kinetic curves (signal growth at 317 nm) shows that the exponential function poorly describes the observed kinetics, while the use of the bimolecular function (Equation (8)) gives a good fit for all measured curves:

(8) $$OD=\frac{{\left[C2\right]}_{0}\varepsilon_{ESSE}}{2}(1-\frac{1}{1+2k_{3obs}{\left[C2\right]}_{0}t})$$

where k_3obs_ is the observed rate constant of the second-order reaction.

The results of the second-order fitting are shown in Figure S4. For all traces, the value of ε_ESSE_ = 6 × 10^3^ M^−1^cm^−1^ was taken, and the value k_3obs_×[C2]_0_ increased from 5.8 × 10^−3^ s^−1^ (for the lowest ESH concentration) to 2.8 × 10^−2^ s^−1^ (for the highest ESH concentration).

We found that the reaction of ESH oxidation and ESSE formation in phosphate buffer proceeds more slowly than in MES buffer. This allows the use of a significantly larger excess of ESH. Figure S5 shows the kinetic curves obtained in 50 mM phosphate buffer (pH 7.0) upon mixing 2 × 10^−4^ M ESH and 1 × 10^−5^ M, 2 × 10^−5^ M, 3 × 10^−5^ M, and 4 × 10^−5^ M CuCl_2_. The perfect agreement with the second-order law even at a 20-fold excess of ESH indicates that under all conditions the ESH oxidation by Cu(II) ions occurs via the bimolecular reactions of C2 complexes.

#### 3.2.2. Influence of Buffer on Observed Second-Order Rate Constant

**Figure 4:**
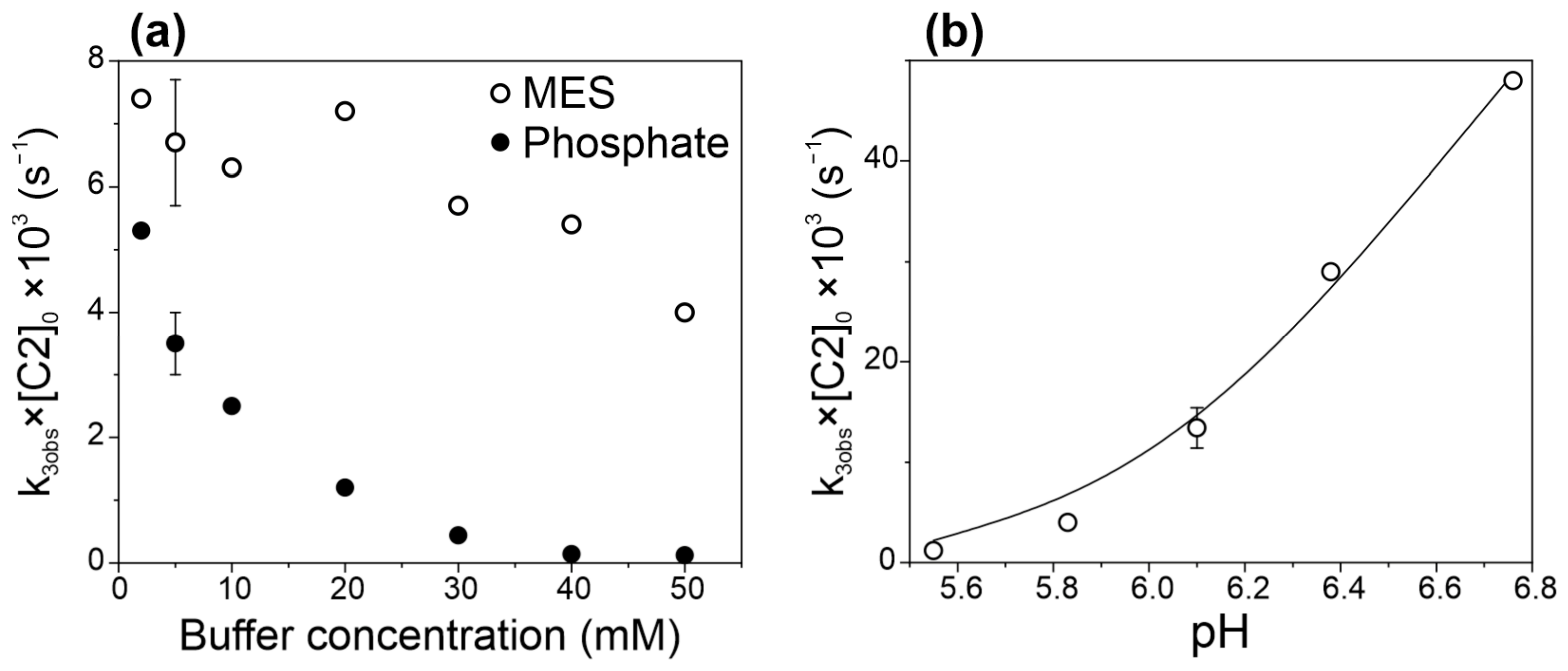
(**a**) The dependence of the rate k_3obs_×[C2]_0_ of ESSE formation on buffer concentration. Empty circles—in MES buffer (pH 6.0), filled circles—in phosphate buffer (pH 7.0). Whiskers show standard deviations measured at a buffer concentration of 5 mM in triplicate. (**b**) The dependence of the rate k_3obs_×[C2]_0_ of ESSE formation in 10 mM MES buffer on pH. Whiskers show standard deviation measured at pH 6.1 in triplicate. Solid line shows the best fit obtained from solving Equations (9)–(11) using K_12_ = K_1_ × K_2_ as the fitting parameter to calculate [C2]_0_ and k_3obs_ as the scaling factor.

The components of phosphate buffer, mainly HPO_4_^2−^ or PO_4_^3−^, can form complexes or even insoluble precipitates with Cu(II) ions [40,41], which can distort the results of the kinetic measurements. In particular, some of the Cu(II) ions can initially be captured in Cu^II^(HPO_4_^2−^) complexes, which significantly reduces the initial concentration of reactive C2 complexes and slows down reaction (6) of ESH oxidation. To test this effect, we performed optical kinetic measurements of ESSE formation at different phosphate buffer concentrations ranging from 2 mM to 50 mM, pH 7.0. The initial concentrations of ESH and CuCl_2_ were 4 × 10^−5^ M and 2 × 10^−5^ M, respectively. The fitting of the initial parts of the kinetic curves to the bimolecular function was conducted using the absorption coefficient of ESSE at 317 nm ε_ESSE_ = 6 × 10^3^ M^−1^cm^−1^ as a constant, while the product of the rate constant k_3obs_ and the initial concentration of C2 complexes k_3obs_×[C2]_0_ was the fitting parameter. The results presented in Figure 4a indicate that indeed, increasing the buffer concentration strongly decelerates ESH oxidation and ESSE formation: with an increase in the buffer concentration from 2 mM to 50 mM, the value of the parameter k_3obs_×[C2]_0_ decreases by approximately 40 times.

It has been reported [41,42] that Cu(II) ions form complexes with most other buffers used in chemical and biological studies, while MES buffer does not complex well with copper. We performed similar measurements of the dependence of the k_3obs_×[C2]_0_ parameter on buffer concentration using MES buffer instead of phosphate buffer; the buffer concentrations ranged from 2 mM to 50 mM, pH 6.0. We found that the observed value k_3obs_×[C2]_0_ almost does not depend on the MES concentration (Figure 4a), and thus, for quantitative kinetic measurements, MES is a much more suitable buffer than phosphate solution. For this reason, most of the experiments were performed with the use of MES buffer.

#### 3.2.3. pH Dependence of the ESH Oxidation Rate

Figure 3c shows the kinetic curves obtained at 317 nm after mixing 4 × 10^−5^ M ESH and 2 × 10^−5^ M CuCl_2_ in aqueous MES solutions (5 mM) with pH 5.5, 5.8, 6.1, 6.4, and 6.7. Increasing the pH causes a significant acceleration of both the signal decay at 257 nm and the signal growth at 317 nm. This confirms that the chemically active species is the anion ES^−^, the concentration of which decreases under acidic conditions (pK_a_ value of ESH is 10.5 [43]).

All kinetic curves obtained at different pHs can be well fitted by the bimolecular function (Equation (8)) with the value k_3obs_×[C2]_0_ increasing from 1.2 × 10^−3^ s^−1^ at pH 5.5 to 4.8 × 10^−2^ s^−1^ at pH 6.7. The obtained values of k_3obs_×[C2]_0_ are shown in Figure 4b by circles. The initial concentration of the C2 complex [C2]_0_ formed immediately after reactant mixing is determined by the following parameters: the initial concentrations of [Cu(II)]_0_ and [ESH]_0_ in solution, the pK_a_ value of ESH, and the stepwise formation constants K_1_ and K_2_. We can calculate [C2]_0_ in accordance with the following equations describing reactions (1), (4) and (5):

(9) $$K_{a}=\frac{\left[H^{+}\right][ES^{-}]}{\left[ESH\right]},$$

(10) $$K_{1}=\frac{\left[C1\right]}{\left[ES^{-}\right][Cu^{2+}]},$$

(11) $$K_{2}=\frac{\left[C2\right]}{\left[ES^{-}\right][C1]}.$$

The obtained pH dependence of the oxidation rate allows us to estimate the value of K_12_ = K_1_ × K_2_. The experimentally observed rate of ESSE formation is assumed to be proportional to [C2]_0_, which in turn is expressed through K_12_ from the equilibrium relations (9–11) and mass balance equations (Supplementary File S1). We fitted the pH dependence of k_3obs_×[C2]_0_ (Figure 4b) for every given pH value using K_12_ as a fitting parameter and k_3obs_ as a scaling factor. The best fit shown in Figure 4b by the solid line was obtained with K_12_ = 1.2 × 10^17^ M^−2^. For simplicity, we assumed that both formation constants are equal, R = K_1_/K_2_ = 1; however, the K_12_ value changes only slightly with the variation of the R value: for example, for R = 10 the best fit was obtained with K_12_ = 1.6 × 10^17^ M^−2^.

### 3.3. Kinetic NMR Measurements

Optical kinetic measurements allow for the unobstructed observation of events occurring only during the first hour after mixing Cu(II) and ESH. To gain insight into the events occurring on a longer timescale, we performed kinetic NMR measurements. To this end, 185 µM CuCl_2_ and 370 µM ESH were mixed in deuterated MES buffer (5 mM, pH 6.0), the NMR sample tube with the mixture was placed into the NMR probehead, and the NMR spectra were recorded every 15 min for 21 h. Figure S6 shows the aromatic regions of the NMR spectra obtained 21 h after mixing. For convenience, we analyzed only the aromatic region of the NMR spectra, since the target signals from the ESH products in the aliphatic region overlap significantly with the signals from the MES protons.

**Figure 5:**
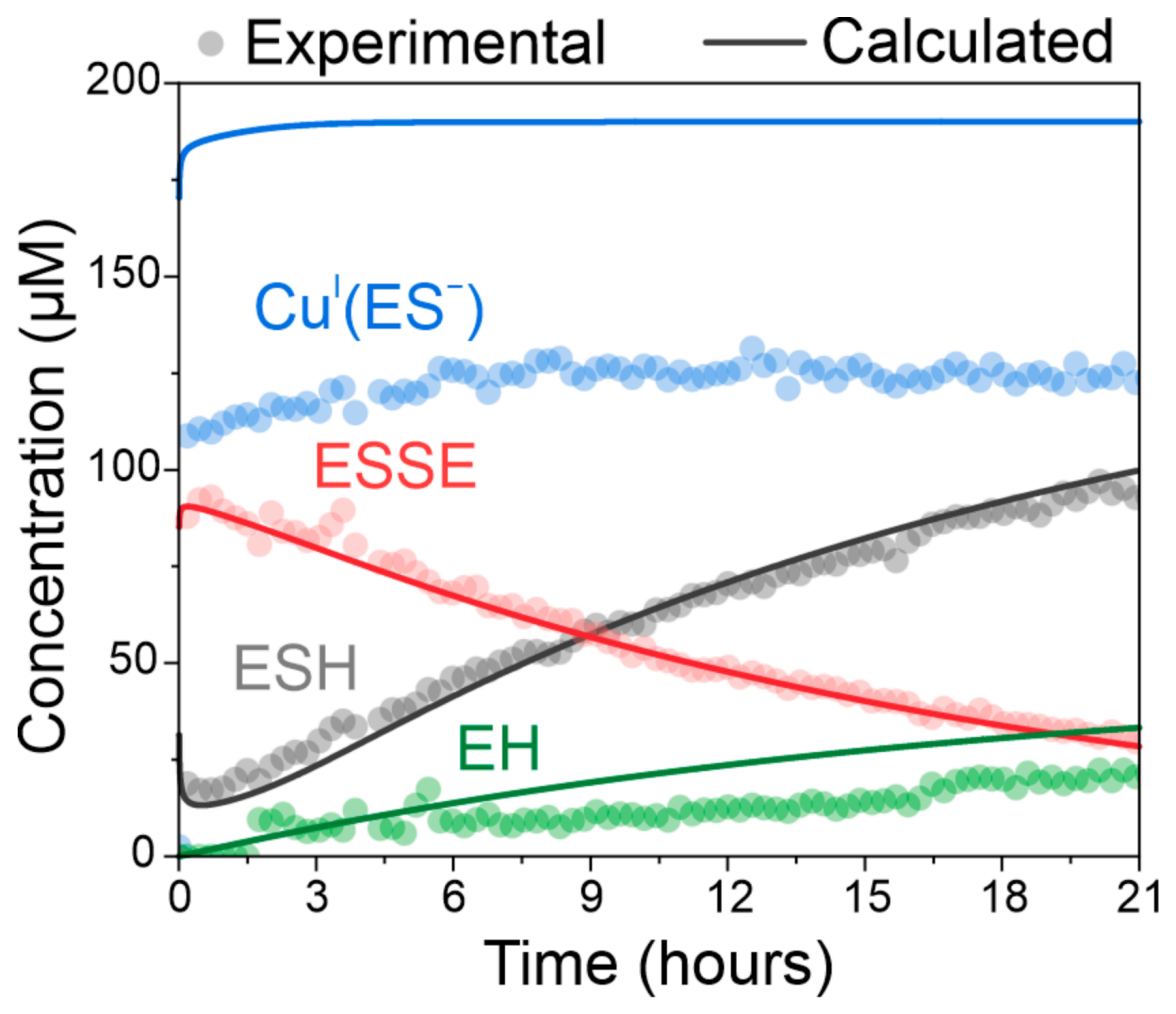
The kinetics of identified reactants detected by ^1^H NMR after mixing 1.85 × 10^−4^ M CuCl_2_ and 3.7 × 10^−4^ M ESH in deuterated MES buffer solution (5 mM, pH 6.0, circles). Solid lines show simulations of ESH, ESSE, EH and C3 formation according to the reaction scheme (1, 4–7) with the following parameters: pH 6.0; [Cu(II)]_0_ = 1.85 × 10^−4^ M; [ESH]_0_ = 3.7 × 10^−4^ M; K_12_ = 1.2 × 10^17^ M^−2^; R = 1; k_3_ = 7.2 × 10^4^ M^−1^s^−1^; k_4_ = 1.7 × 10^−5^ s^−1^.

As expected, the products of the reaction are ESH, Cu^I^(ES^−^) complex, ESSE, EH (deuterated at C6 position of the imidazole ring) and several minor unrecognized compounds. Unrecognized signals in the NMR spectra are annotated as S###, where S stands for the singlet, and ### corresponds to the signal chemical shift: for example, the signal at 6.77 ppm is annotated as S677. Among the unrecognized signals, S665 and S677 have kinetics that are very similar to that of the C3 complex (6.55 ppm), while the kinetics of S720 is similar to that of EH (7.15 ppm). Therefore, the signals at 6.65 ppm and 6.77 ppm may correspond to different configurations of the Cu(I) and ES^−^ complex. The S720 signal probably corresponds to an unknown product of ESSE hydrolysis. Apparently, further structural studies are required to confirm the suggested attribution of signals S665, S677 and S720. Figure 5 illustrates the long-term kinetics of ESH and the major products of the reaction. The initial compound, ESH, demonstrates a decay during the first hour of the reaction, and then its concentration increases, reaching approximately 30% of its initial concentration. In contrast, the concentration of ESSE first increases and then decreases. The C3 complex forms immediately after mixing CuCl_2_ and ESH and then demonstrates a small additional increase within the first five hours. The concentration of EH slowly increases during the whole experiment, reaching 20 μM by the end of the experiment, which is about 7% of the initial ergothioneine concentration. At the end of the experiment (21 h after the beginning of the reaction), the following concentrations of reagents have been reached: ESH—100 µM; ESSE—30 µM; Cu^I^(ES^−^) complex—120 µM; and EH—20 µM. It should be noted that 21 h after the mixing, the reaction was not fully completed, and further decomposition of ESSE and the formation of ESH and EH is expected. The initial ESH concentration was 370 µM, while the total concentration of identified ESH-derived products reached 300 µM, meaning that about 80 µM (~21%) of the ESH products remained unidentified. To verify the reproducibility of the measurements, the kinetic experiment using the NMR method was repeated; both experiments yielded virtually identical results.

### 3.4. Modeling of the Kinetic Scheme

To gain mechanistic insight into the interaction of ESH and Cu(II) and to extract kinetic parameters from the experimental data, we performed model calculations. The kinetic model takes into account the following processes: the deprotonation of ESH to ES^−^ (reaction (1)), the stepwise formation of mono- (C1) and bis-ligand (C2) Cu(II) complexes with ES^−^ (reactions (4,5)), the bimolecular reaction of two C2 complexes yielding the disulfide product ESSE and Cu(I) complex C3 (reaction (6)), and ESSE hydrolysis (reaction (7)).

In fact, reaction (7) consists of several steps, including the formation of sulfenic ESOH and sulfinic ESO_2_H acids [27]. However, we did not observe the formation of ESOH and ESO_2_H either by LC-MS or NMR measurements. This indicates that the reactions of ESOH and ESO_2_H are fast, and the rate of formation of the final reaction products ESH and EH is determined by the first-order rate constant k_4_ of ESSE hydrolysis. Reactions (1), (4), and (5) are assumed to be much faster than reactions (6) and (7). This timescale separation allows us to use a partial equilibrium approximation, where the concentrations of ESH, ES^−^, C1, C2, and Cu(II) are considered to be in equilibrium according to relations (9–11), while the dynamics of the slowly changing variables [C2], [ESSE], [C3], [ESH] and [EH] are described by kinetic differential equations:

(12) $$\frac{d[C2]}{dt}=-2k_{3}{\left[C2\right]}^{2},$$

(13) $$\frac{d[C2]}{dt}=-2k_{3}{\left[C2\right]}^{2},$$

(14) $$\frac{d\left[ESSE\right]}{dt}=k_{3}{\left[C2\right]}^{2}-k_{4}[ESSE],$$

(15) $$\frac{d\left[ESH\right]}{dt}={1.5k}_{4}[ESSE],$$

(16) $$\frac{d\left[EH\right]}{dt}={0.5k}_{4}[ESSE].$$

Since reactants C2 and ESH participate in both fast and slow reactions, the resulting mixed algebraic–differential system does not have a convenient analytical solution. Therefore, in our model, this system of equations was solved iteratively by alternating the kinetic evolution of C2, C3, ESSE, ESH and EH with the re-establishment of equilibration of the fast subsystem. A detailed description of the solution of the differential–algebraic equation system is given in the SI (Supplementary File S1).

The model parameters include: [H^+^], determined by the buffering system and measured in each experiment; the initial concentrations [ESH]_0_ and [Cu(II)]_0_, which were fixed parameters; and the ESH acidity constant K_a_ = 3.16 × 10^−11^ (pK_a_ = 10.5) [43]. The stepwise stability constants K_1_ and K_2_ were considered equal (R = 1), and the value K_12_ = K_1_ × K_2_ = 1.2 × 10^17^ M^−2^ was estimated from the pH dependence of the ESH oxidation rate (Section 3.2.3.). The rate constants k_3_ and k_4_ for reactions (6) and (7) were considered as adjustable parameters and were determined from the fitting of the calculated kinetic curves to the experimental optical and NMR data. The value k_4_ = (1.7 ± 0.4) × 10^−5^ s^−1^ was determined from the NMR kinetic measurements (two replicates), while the rate constant k_3_ was the only fitting parameter in the simulation of optical kinetic curves obtained at 317 nm. For all kinetic optical (a total of nine kinetic curves at various ESH concentrations and pH values) and NMR (two replicates under identical conditions) data, a fairly good agreement was obtained with the same set of parameters mentioned above, with k_3_ values ranging from 5.4 × 10^4^ M^−1^s^−1^ to 1.3 × 10^5^ M^−1^s^−1^. The results of the calculations are shown in Figure 3b,c and Figure 5 by the solid lines.

## 4. Discussion

**Scheme 1:**
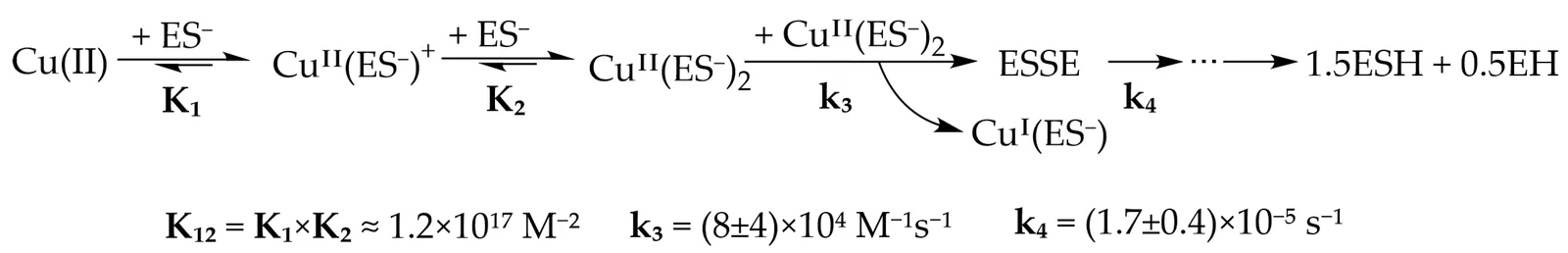
Scheme describing the mechanism of Cu(II) reduction by ESH followed by ESSE decomposition in aqueous solution. The dots ••• denote intermediate steps of the reaction described below by Equations (18)–(20).

The present work is the first comprehensive study of the interactions between the antioxidant ESH and Cu(II) ions in aqueous solutions. To study the various stages of the reaction, we used several modern physical and chemical methods such as LC-MS, NMR, and spectrophotometry. We obtained a large set of kinetic and steady-state spectral data, which allowed us to identify and characterize the intermediate and final reaction products, as well as to measure the kinetic characteristics of the reactions studied, and finally to propose a detailed mechanism for these reactions (Scheme 1).

The initial stage of the reaction occurring on a subsecond time scale immediately after mixing the reactants is the formation of complexes C1 (Cu^II^(ES^−^)^+^) and C2 (Cu^II^(ES^−^)_2_). The initial concentrations of substances in solution, including ESH, ES^−^, Cu(II), C1, and C2, are determined by equilibria (1, 4–5) and depend on the reagent concentrations, the pH of the solution and the complex formation constants K_1_ and K_2_. Bimolecular reaction (6) of the C2 complexes yields ESSE and C3 (Cu^I^(ES^−^)) as intermediate and final reaction products, respectively. The observed rate of this reaction depends on the bimolecular rate constant k_3_ and the initial concentration of C2. Using the dependence of the observed reaction rate on the pH, we estimated the value of the complexation constant K_12_ = K_1_ × K_2_ as 1.2 × 10^17^ M^−2^. This value is noticeably lower than that reported by Hanlon [32] (K_12_ = 1.6 × 10^18^ M^−2^) and by Motohashi et al. [43] (K_12_ = 8 × 10^18^ M^−2^). One should take into account that in these works [32,43], the formation constant K_12_ was measured using the titration method without taking into account the bimolecular reaction of the Cu^II^(ES^−^)_2_ complexes. This reaction leads to the oxidation of ESH and the formation of the Cu^I^(ES^−^) complex and therefore significantly affects the results of the steady-state titration.

The rate of ESH oxidation by Cu(II) ions significantly depends on the buffer system used in the experiments. According to Kotuniak et al. [41], MES is one of the few buffers that does not form stable complexes or insoluble precipitates with Cu(II). Most of the other frequently used buffers, including phosphate, interact with Cu(II) ions, which reduces the concentration of available free Cu(II) ions in solution [42]. We found that the rate of ESSE formation in 50 mM MES buffer at pH 6.0 is approximately 30-fold higher than that in 50 mM phosphate buffer at pH 7.0 (Figure 4a). Since the decrease in pH causes a decrease in the observed reaction rate (Figure 4b), one can assume that at equal pH values, the difference between MES and phosphate would be even greater. To take the effect of buffer into account, in model calculations, we added an additional equilibrium (17) to the set of reactions (1, 4–5):

(17) $$\mathrm{Cu}(\mathrm{II})+{\mathrm{HPO}}_{4}2^{-}\;⇄\mathrm{C4},\to K_{\mathrm{phos}}$$

where C4 is the complex Cu^II^(HPO_4_^2−^). The solution of equations describing reactions (1,4–5,17) was performed with the following parameters: [ESH]_0_ = 4 × 10^−5^ M, [Cu(II)]_0_ = 2 × 10^−5^ M, 50 mM phosphate buffer, pH 7.0, pK_a_(ESH) = 10.5, K_12_ = 1.2 × 10^17^ M^−2^, R = 1, and K_phos_ = 1.6 × 10^3^ M^−1^ [44]. The calculations showed that the presence of 50 mM phosphate does indeed reduce the initial concentration of complex C2, but only by half, which is obviously insufficient to explain the huge difference in the reaction rates in phosphate and MES buffers. This likely indicates that phosphates coordinate not only Cu(II) ions but also C1 and C2 complexes, reducing their reactivity and decreasing the rate of reaction of ESH oxidation. Our findings highlight the importance of choosing a suitable buffer system when studying reactions with metal ions.

The bimolecular reaction between C2 complexes yields one stable product, C3 complex, and one unstable product, ESSE. The mechanism of ESSE decomposition in aqueous solution was previously studied by Servillo et al. [27] and includes the formation of intermediate sulfenic ESOH and sulfinic ESO_2_H acids, the restoration of reduced ergothioneine ESH, and the formation of the final reaction product hercynine EH:

ESSE + H_2_O → ESOH + ESH, (18)

2ESOH → ESO_2_H + ESH, (19)

ESO_2_H + H_2_O → EH + H_2_SO_3_. (20)

The decomposition of ESSE and the formation of the reaction products were monitored by kinetic ^1^H NMR (Figure 5). We found that at 25 °C, the rate constant of the ESSE decomposition is k_4_ = (1.7 ± 0.4) × 10^−5^ s^−1^.

All kinetic data obtained in this work, including the dependence of the ESSE formation kinetics on the initial concentrations of the reactants (Figure 3b) and on the pH (Figure 3c), as well as the kinetics of transformation of substances detected in kinetic NMR measurements (Figure 5), were modeled using the same set of parameters. The only variable parameter was the rate constant k_3_ of the bimolecular reaction (6) of C2 complexes. For all optical kinetic curves, good agreement between calculated and experimental data was obtained. In the calculations of the kinetic curves of ESSE formation for different initial ESH concentrations (Figure 3b), the value of k_3_ varied from 5.5 × 10^4^ M^−1^s^−1^ to 7.8 × 10^4^ M^−1^s^−1^, whereas in the calculations for different pH values (Figure 3c), k_3_ varied from 7.2 × 10^4^ M^−1^s^−1^ to 1.3 × 10^5^ M^−1^s^−1^. The average value of k_3_ obtained from all optical kinetic measurements (nine kinetic curves) is k_3_ = (8 ± 4) × 10^4^ M^−1^s^−1^.

The NMR kinetic data show very good agreement between the calculations and experiment for ESH and ESSE. However, the calculated curves for EH and the C3 complex demonstrate only qualitative agreement with the experimental data (Figure 5) and indicate an insufficient amount of these substances formed in the experiment. In the aromatic region of the NMR spectra, we found two signals at 6.65 ppm and 6.77 ppm, the kinetics of which are very similar to the kinetics of C3. We also observed the same signals at 6.65 ppm and 6.77 ppm in the NMR spectrum of the C3 complex obtained by mixing ESH with CuCl. Therefore, it can be speculated that these signals correspond to different forms of the complex of ESH with Cu(I) ions, such as the binding of Cu(I) ions to different sites of ESH or the formation of complexes with different stoichiometry [39]. One more unrecognized signal, S720 (Figure S7), probably corresponds to an unknown product of ESSE hydrolysis. Taking into account the contribution of the compounds related to signals S665, S677, and S720 significantly improves the balance between the initial concentration of ESH and the final concentrations of all reaction products.

We should note that the quantitative simulations performed in the present work within the framework of the reaction scheme (1), (4)–(7) have some limitations:

1. We assumed that only C2 complexes undergo bimolecular reactions. However, C1 complexes can also react, for example, in the reactions

C1 + C2 → C3 + ESSE + Cu(I),(21)

and

C1 + C1 → 2Cu(I) + ESSE(22)

Taking these reactions into account may reduce the calculated value of the rate constant k_3_.

2. We assumed that the bimolecular reaction between C2 complexes is independent of the pH of the solution and the pK_a2_ value of the C2 complex (Supplementary File S1, Equation (15)). This assumption is true if the reactivity of the C2 complex does not depend on its protonation state, or if the pK_a2_ value of complex C2 is lower than all the pH values of the solutions used in this work, and if the C2 complexes are predominantly in the deprotonated state. This seems to be quite reasonable since the binding of copper to ESH significantly reduces the pK_a_ value: in particular, for complex C3, a pK_a_ value of approximately 5.0 can be obtained from the data presented by Motohashi et al. [33]. We tried to simulate the observed optical kinetics and their pH dependence using pK_a2_ values in the range of 6–8, but this did not improve the fitting results.

The results obtained in this work demonstrate the advantages of ESH over other antioxidants (such as GSH [45,46,47]) in protecting living cells against oxidative damage induced by Cu(II) ions. First, ESH reduces Cu(II) ions to less toxic (weaker oxidizing agent) Cu(I) ions without generating radicals or other highly reactive species. The resulting Cu(I) ions remain bound in stable C3 complexes, which prevents their participation in pro-oxidant cycles [35]. It is noteworthy that under aerobic conditions, the simultaneous presence of H_2_O_2_ or O_2_^•−^ and redox-active metals can lead to the formation of highly damaging hydroxyl radicals and other reactive oxidants via Fenton and Haber–Weiss reactions [48,49,50]. This dual protecting function of ESH is supported by the work of Zhu et al. [35], who showed that ESH effectively protects DNA and proteins from copper-induced oxidative degradation even in the presence of H_2_O_2_, implying that the chelated Cu(I) species is redox-inactive. Furthermore, in the presence of metal ions, GSH and ascorbate can be oxidized by molecular oxygen to yield superoxide [51,52,53], whereas ESH does not undergo this process. Moreover, the hydrolysis of ESSE regenerates the reduced form of ESH without the involvement of reducing agents such as NADH [27], unlike GSH or ascorbate.

The total balance of the proposed reaction scheme is

8ESH + 4Cu(II) +3H_2_O → 4Cu^I^(ES^−^) + 3ESH + EH + H_2_SO_3_. (23)

Taking into account that ESH in C3 complexes does not form covalent bonds and that ESH molecules remain unchanged, the conversion of one ESH molecule into EH reduces four Cu(II) ions, which remain bound in chemically stable Cu^I^(ES^−^) complexes. Further removal of C3 from the body might include the complex dissociation and release of free ESH. Servillo et al. [27] remarked that in a biological system, the oxidation of one ESH molecule to EH and sulfate involves the transfer of six electrons to intracellular oxidants, making ESH a rich source of reducing equivalents, which are then slowly released into the cell and provide protection against oxidative stress. An additional advantage of ESH is that, being an exogenous antioxidant, it does not require resources for its synthesis.

The biological role of EH formed during the redox reaction is largely unexplored. A previous metabolomic study [12] showed that avian lenses contain large amounts of ESH, while EH was not detected. We also found no reports on significant EH concentrations in blood plasma. Therefore, it can be assumed that EH is excreted from cells through the membrane and then eliminated from the body.

The proposed reaction scheme explains most of the previous observations on the protective role of ESH against copper-induced oxidative damage. In particular, millimolar concentrations of ESH completely inhibit deoxyribose degradation in the presence of Cu(II) ions [24]. The addition of ESH inhibits the Cu(II)-catalyzed oxidation of ascorbate, the formation of carbonyl groups in proteins, and DNA strand break formation [35]. Ey et al. [36] demonstrated the protective effect of ESH against Cu(II)-induced cytotoxicity in OCTN1-expressing HEK-293 cells but not in HEK-293 cells lacking OCTN1. All these effects can be explained by the reduction of divalent copper to the Cu(I) state and the binding of the resulting Cu(I) ions to ergothioneine to form stable C3 complexes.

## 5. Conclusions

In this work, we established a detailed mechanism of the interactions of ergothioneine with Cu(II) ions in aqueous solutions. When copper ions enter a solution containing ESH, the complexes C1 (Cu^II^(ES^−^)^+^) and C2 (Cu^II^(ES^−^)_2_) are formed first. The bimolecular reaction between the C2 complexes proceeds with a rate constant k_3_ and leads to the reduction of Cu(II) in complex to Cu(I) bound in the stable C3 complex (Cu^I^(ES^−^)) and ESH oxidation to form ESSE disulfide. The value of k_3_ can be estimated as (8 ± 4) × 10^4^ M^−1^s^−1^. The use of phosphate buffers significantly slows the rate of ESH oxidation due to the complexation of Cu(II) ions and, most likely, the C1 and C2 complexes with phosphates. The rate of ESH oxidation by Cu(II) ions strongly depends on the pH of the solution: as the pH decreases, the initial concentrations of ES^−^ ions and hence the C2 complex decrease, which leads to a decrease in the observed rate of the bimolecular reaction of the C2 complexes. It is important to note that after the redox reaction, Cu(I) ions remain bound in stable complexes with ES^−^, which prevents the generation of reactive oxygen species.

ESSE disulfide in aqueous solutions undergoes spontaneous hydrolysis with a rate constant of (1.7 ± 0.4) × 10^−5^ s^−1^, which regenerates reduced ESH and yields the final reaction product, hercynine EH. According to the reaction scheme proposed, the conversion of one ESH molecule to EH causes the reduction of four Cu(II) ions. From a biological point of view, the advantages of ESH in deactivating toxic Cu(II) ions (reducing Cu(II) to the less toxic Cu(I) state with high efficiency and without generating radicals, chelating the formed Cu(I) ions) compared to other antioxidants indicate that cellular protection against oxidation by metal ions is likely one of the main functions of ESH. This justifies the high ESH abundance in tissues that are most susceptible to oxidative stress.

## Data Availability

The original contributions presented in this study are included in the article/Supplementary Materials. Further inquiries can be directed to the corresponding author.

## Supplementary Materials

The following supporting information can be downloaded at https://www.mdpi.com/article/10.3390/antiox15091064/s1, Figure S1: MS/MS fragmentation spectra of the parent ion 230.0958; Figure S2: MS/MS fragmentation spectra of the parent ion 457.1680; Figure S3: MS/MS fragmentation spectra of the parent ion 198.1236; Figure S4: Second-order fitting of kinetics of optical absorption monitored at 317 nm after the mixing of 2 × 10^−5^ M CuCl_2_ and various concentrations of ESH in MES buffer (5 mM, pH 6.0); Figure S5: Second-order fitting of kinetics of optical absorption detected at 317 nm after the mixing of various concentrations of CuCl_2_ and 2 × 10^−4^ M ESH in phosphate buffer (50 mM, pH 7.0); Figure S6: Aromatic region of ^1^H NMR spectrum obtained 21 h after mixing 3.7 × 10^−4^ M ESH and 1.85 × 10^−4^ M CuCl_2_ in deuterated MES buffer (5 mM, pH 6.0); Figure S7: Base peak chromatograms from LC-MS experiment obtained before and 24 h after mixing 2 × 10^−4^ M CuCl_2_ and 4 × 10^−4^ M ESH in MES buffer (5 mM, pH 6.0); Supplementary File S1: Model of ESH oxidation by Cu(II) ions.
