# Supplementary material for: Mechanism of Cu(II) Detoxification by Ergothioneine: Reduction and Formation of Stable Cu(I) Complexes
Source: Antioxidants (Basel). 2026 Aug 25;15(9):1064. doi: 10.3390/antiox15091064 (PMC13603105; doi:10.3390/antiox15091064)
Supplement: Supplementary file 1 [file antioxidants-15-01064-s001.zip › antioxidants-4481758-supplementary.pdf]

Supplementary Information

for

Mechanism of Cu(II) Detoxification by Ergothioneine: Reduction and Formation of Stable Cu(I) Complexes

Yuri P. Tsentalovich *, Nataliya A. Osik, Maxim V. Fomenko, Nikita A. Dmitriev and Vadim V. Yanshole

Laboratory of Proteomics and Metabolomics, International Tomography Center SB RAS, Institutskaya Street 3a, 630090 Novosibirsk, Russia; n.osik@tomo.nsc.ru (N.A.O.); m.fomenko@tomo.nsc.ru (M.V.F.); n.dmitriev1@g.nsu.ru (N.A.D.); vadim.yanshole@tomo.nsc.ru (V.V.Y.)

* Correspondence: yura@tomo.nsc.ru

Table of contents

Figure S1. MS/MS fragmentation spectra of the parent ion 230.0958.

Figure S2. MS/MS fragmentation spectra of the parent ion 457.1680.

Figure S3. MS/MS fragmentation spectra of the parent ion 198.1236.

Figure S4. Second-order fitting of kinetics of optical absorption monitored at 317 nm after the mixing of 2×10^{-5} M $CuCl_2$ and various concentrations of ESH in MES buffer (5 mM, pH 6.0).

Figure S5. Second-order fitting of kinetics of optical absorption detected at 317 nm after the mixing various concentrations of $CuCl_2$ and 2×10^{-4} M ESH in phosphate buffer, (50 mM, pH 7.0).

Figure S6. Aromatic region of ^{1}H NMR spectrum obtained 21 hours after mixing 3.7×10^{-4} M ESH and 1.85×10^{-4} M $CuCl_2$ in deuterated MES buffer (5 mM, pH 6.0).

Figure S7. Base peak chromatograms from LC-MS experiment obtained before and 24 hours after mixing 2×10^{-4} M $CuCl_2$ and 4×10^{-4} M ESH in MES buffer (5 mM, pH 6.0).

Supplementary File S1. Model of ESH oxidation by Cu(II) ions.

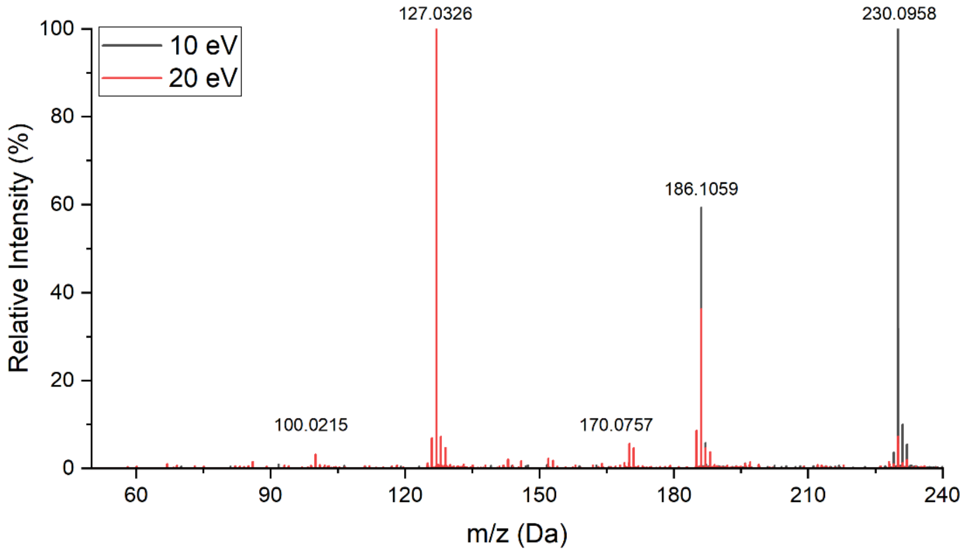

Figure S1. MS/MS fragmentation spectra of the parent ion 230.0958 eluted at RT=3.80 min during LC separation of the reaction mixture 24 hours after mixing 2×10^{-4} M $CuCl_2$ and 4×10^{-4} M ESH in MES buffer (5 mM, pH 6.0) obtained with CID energy 10 eV (black) and 20 eV (red).

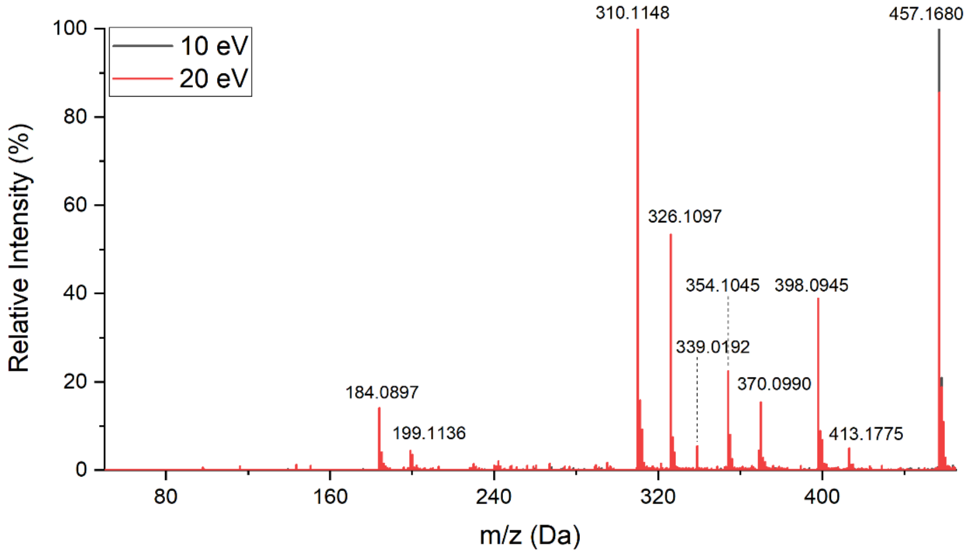

Figure S2. MS/MS fragmentation spectra of the parent ion 457.1680 eluted at RT=10.10 min during LC separation of the reaction mixture 24 hours after mixing 2×10^{-4} M $CuCl_2$ and 4×10^{-4} M ESH in MES buffer (5 mM, pH 6.0) obtained with CID energy 10 eV (black) and 20 eV (red).

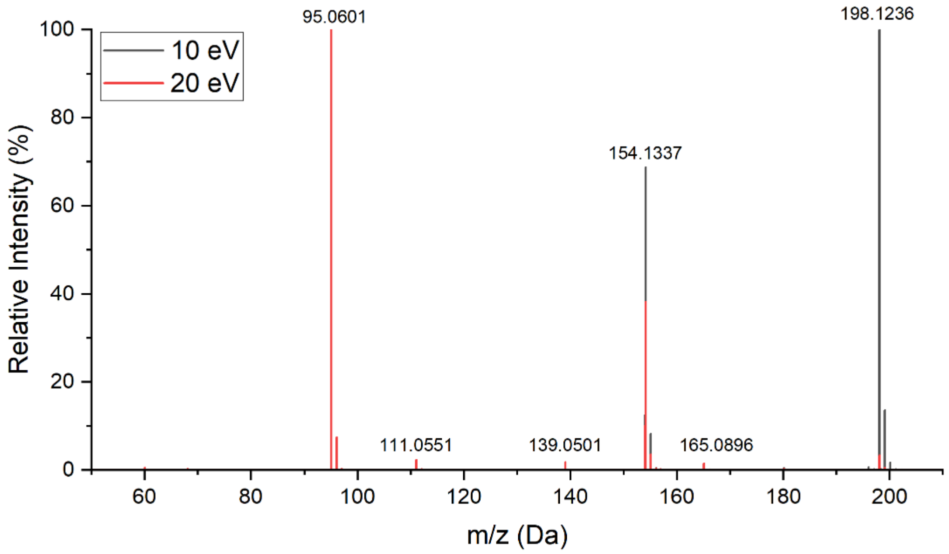

Figure S3. MS/MS fragmentation spectra of the parent ion 198.1236 eluted at RT=3.01 min during LC separation of the reaction mixture 24 hours after mixing 2×10^{-4} M $CuCl_2$ and 4×10^{-4} M ESH in MES buffer (5 mM, pH 6.0) obtained with CID energy 10 eV (black) and 20 eV (red).

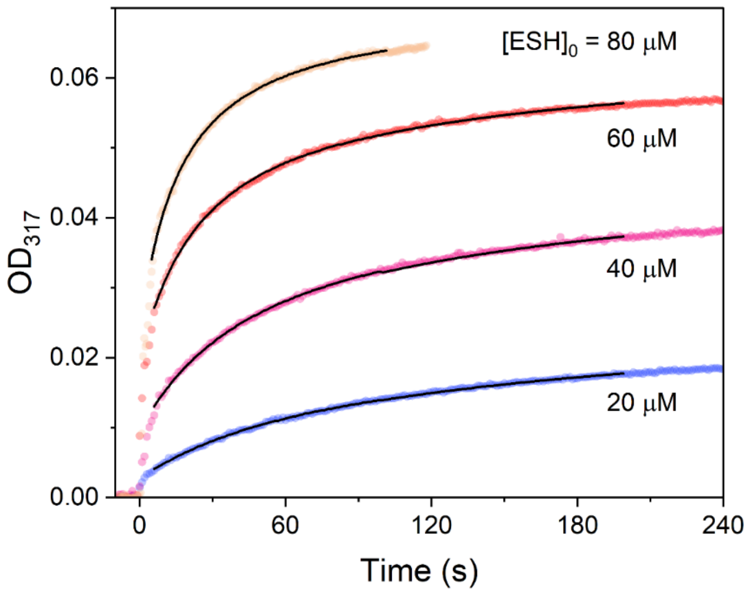

Figure S4. Second-order fitting of kinetics of optical absorption monitored at 317 nm after the mixing of 2×10^{-5} M $CuCl_2$ and various concentrations of ESH in MES buffer (5 mM, pH = 6.0). Concentrations of ESH are indicated on the plot. Black lines show the best second-order fits (Eq. 8 in the main text) obtained with $\varepsilon_{ESSE} = 6\times10^3$ $M^{-1}cm^{-1}$ and $k_{3obs}\times[C2]_0$ increasing from 5.8×10^{-3} s^{-1} (for $[ESH]_0 = 2\times10^{-5}$ M) to 2.8×10^{-2} s^{-1} (for $[ESH]_0 = 8\times10^{-5}$ M).

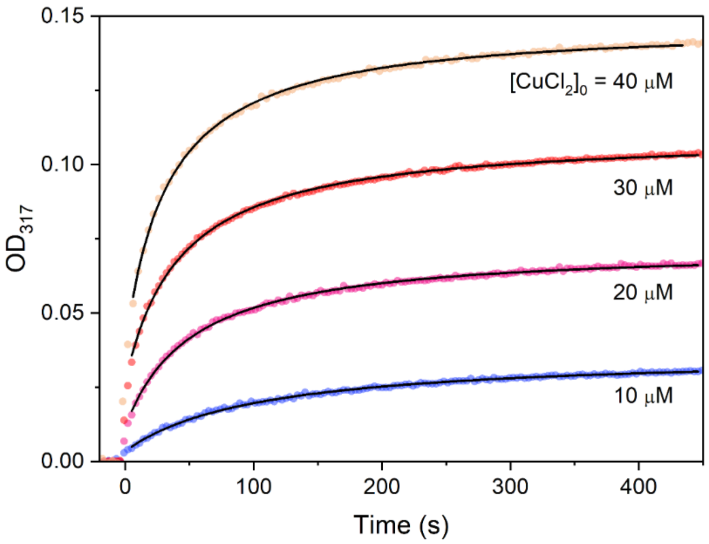

Figure S5. Second-order fitting of kinetics of optical absorption detected at 317 nm after the mixing various concentrations of $CuCl_2$ and 2×10^{-4} M ESH in phosphate buffer, (50 mM, pH 7.0). Concentrations of $CuCl_2$ are indicated on the plot. Black lines show the best second-order fits (Eq. 8 in the main text) obtained with $\varepsilon_{ESSE} = 6\times10^3$ $M^{-1}cm^{-1}$ and $k_{3obs}\times[C2]_0$ increasing from 4.7×10^{-3} s^{-1} for $[CuCl_2]_0 = 1\times10^{-5}$ M to 1.7×10^{-2} s^{-1} for $[CuCl_2]_0 = 4\times10^{-5}$ M.

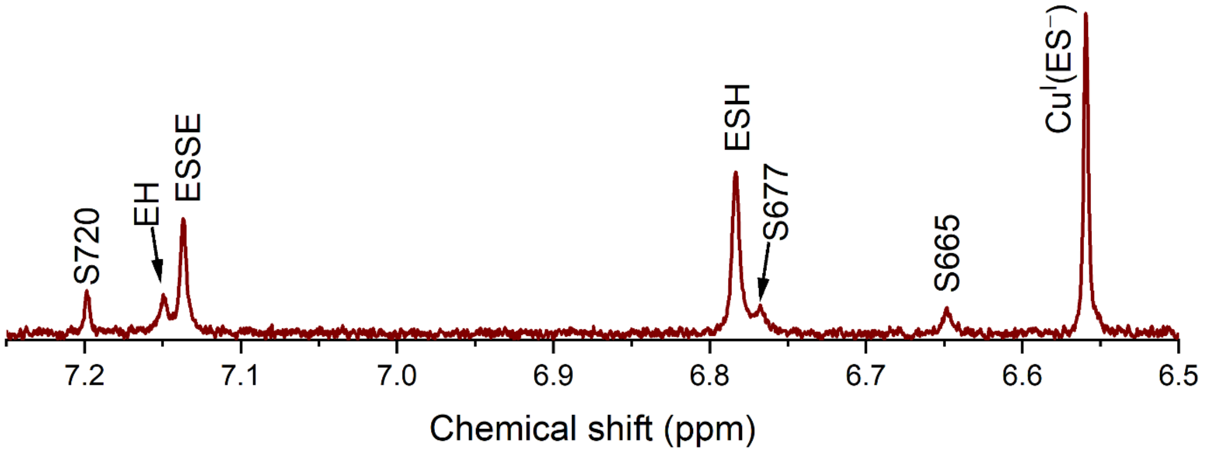

Figure S6. Aromatic region of ^1H NMR spectrum obtained 21 hours after mixing 370 μM ESH and 185 μM $CuCl_2$ in deuterated MES buffer (5 mM, pH 6.0).

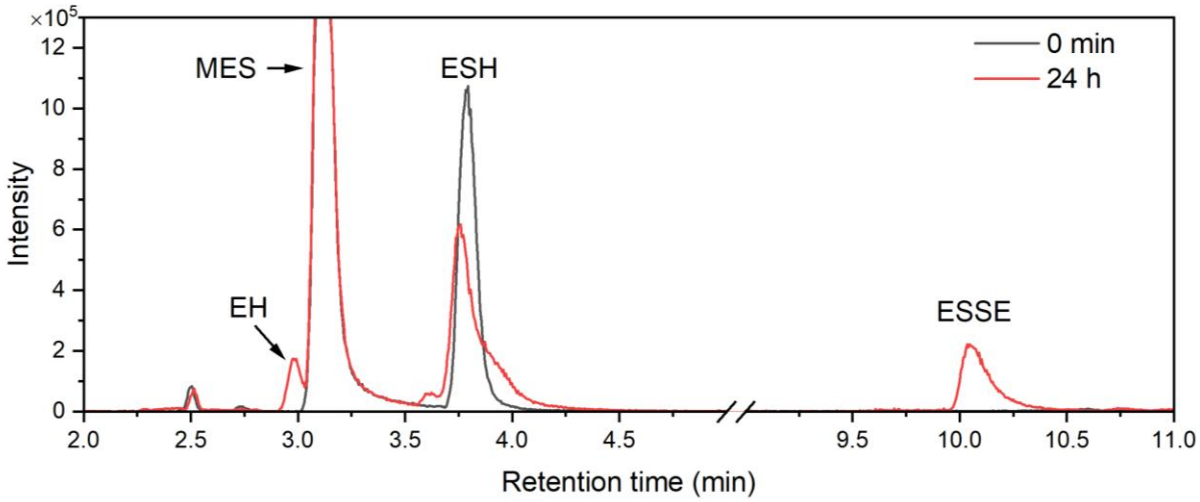

Figure S7. Base peak chromatograms from LC-MS experiment obtained before (black line) and 24 hours after (red line) mixing 2×10^{-4} M $CuCl_2$ and 4×10^{-4} M ESH in MES buffer (5 mM, pH 6.0).

Supplementary File S1. Model of ESH oxidation by Cu(II) ions.

To gain mechanistic insight into the interaction of ESH and Cu(II) and to extract kinetic parameters from experimental data, we performed model calculations. The kinetic model takes into account the following processes: deprotonation of ESH to ES^-, stepwise formation of mono- ($C1 = Cu^{II}(ES^-)^+$) and bis-ligand ($C2 = Cu^{II}(ES^-)_2$) Cu(II) complexes with ES^-, the bimolecular reaction of copper reduction yielding disulfide product ESSE and Cu(I) complex ($C3 = Cu^I(ES^-)$), and ESSE hydrolysis:

$$\mathrm{ESH} \rightleftarrows \mathrm{ES^-} + \mathrm{H^+},\ \mathrm{Ka} \tag{S1}$$

$$\mathrm{Cu^{2+}} + \mathrm{ES^-} \rightleftarrows \mathrm{C1},\ \mathrm{K_1} \tag{S2}$$

$$\mathrm{C1} + \mathrm{ES^-} \rightleftarrows \mathrm{C2},\ \mathrm{K_2} \tag{S3}$$

$$\mathrm{2C2} \longrightarrow \mathrm{ESSE} + \mathrm{2C3},\ \mathrm{k_3} \tag{S4}$$

$$\mathrm{ESSE} \longrightarrow \mathrm{1.5ESH} + \mathrm{0.5EH},\ \mathrm{k_4} \tag{S5}$$

Reactions (S1) – (S3) are assumed to be much faster than the reactions (S4) – (S5). This timescale separation allows us to use a partial equilibrium approximation, when the concentrations of ESH, ES^-, C1 and Cu(II) and C2 are considered to be equilibrium in accordance to the following relations:

$$\mathrm{Ka} = \frac{[\mathrm{H^+}][\mathrm{ES^-}]}{[\mathrm{ESH}]}, \tag{S6}$$

$$\mathrm{K_1} = \frac{[\mathrm{C1}]}{[\mathrm{ES^-}][\mathrm{Cu(II)}]}, \tag{S7}$$

$$\mathrm{K_2} = \frac{[\mathrm{C2}]}{[\mathrm{ES^-}][\mathrm{C1}]}, \tag{S8}$$

while the dynamics of slowly changing variables C2, ESSE, C3, ESH and EH is described by kinetic differential equations:

$$\frac{\mathrm{d[C2]}}{\mathrm{dt}} = -2\mathrm{k_3}[\mathrm{C2}]^2, \tag{S9}$$

$$\frac{\mathrm{d[C3]}}{\mathrm{dt}} = 2\mathrm{k_3}[\mathrm{C2}]^2, \tag{S10}$$

$$\frac{\mathrm{d[ESSE]}}{\mathrm{dt}} = \mathrm{k_3}[\mathrm{C2}]^2 - \mathrm{k_4}[\mathrm{ESSE}], \tag{S11}$$

$$\frac{\mathrm{d[ESH]}}{\mathrm{dt}} = 1.5\mathrm{k_4}[\mathrm{ESSE}], \tag{S12}$$

$$\frac{\mathrm{d[EH]}}{\mathrm{dt}} = 0.5\mathrm{k_4}[\mathrm{ESSE}]. \tag{S13}$$

Since reactants C2 and ESH participate in both fast and slow reactions, the resulting mixed algebraic–differential system does not have a convenient analytical solution.

Therefore, in our model this system of equations was solved iteratively by alternating the kinetic evolution of C2, C3, ESSE, ESH and EH with the re-establishment of equilibration of the fast subsystem.

To account for possible differences between the acidity of free and complexed ESH, we introduced distinct deprotonation constants Ka_1 and Ka_2 for the protonated complexes C1H and C2H.

$$\mathrm{C1H} \rightleftarrows \mathrm{H^+} + \mathrm{C1}, \mathrm{Ka_1} \quad \text{(S14)}$$

$$\mathrm{C2H} \rightleftarrows \mathrm{H^+} + \mathrm{C2}, \mathrm{Ka_2} \quad \text{(S15)}$$

To take the effect of phosphate buffer into account, we added an additional equilibrium:

$$\mathrm{Cu(II)} + \mathrm{HPO_4^{2-}} \rightleftarrows \mathrm{C4}, \mathrm{K_{phos}} \quad \text{(S16)}$$

The model parameters include $[H^+]$ determined by buffering system, initial concentrations $[ESH]_0$ and $[Cu(II)]_0$, ESH acidity constant Ka = 3.16×10^{-11} (pKa = 10.5). The stepwise stability constants K_1 and K_2 were considered equal, and the value of $K_{12}=K_1\times K_2 = 1.2\times10^{17}$ M^{-2} was estimated from the pH-dependence of the ESH oxidation rate (Section 3.2.4.). The rate constants k_3 and k_4 for reactions (S4) and (S5), respectively, were treated as adjustable parameters and determined from kinetic optical and NMR data.

First, the concentrations of the "fast" subsystem components at the initial time moment were calculated. Using mass balance equations

$$[\mathrm{ESH}]_0 = [\mathrm{ESH}] + [\mathrm{ES^-}] + [\mathrm{C1}] + 2[\mathrm{C2}] + [\mathrm{C1H}] + 2[\mathrm{C2H}], \quad \text{(S17)}$$

$$[\mathrm{Cu(II)}]_0 = [\mathrm{Cu(II)}] + [\mathrm{C1}] + [\mathrm{C2}], \quad \text{(S18)}$$

and equilibrium relations (S6) – (S8), we obtained a cubic equation for free ESH concentration with coefficients A, B, C, D expressed in terms of $[H^+]$, $[ESH]_0$, $[Cu(II)]_0$, Ka, K_1, K_2, K_{12}, Ka_1, Ka_2:

$$\mathrm{A[ESH]^3 + B[ESH]^2 + C[ESH] + D = 0,}$$

$$\mathrm{A} = \mathrm{K_{12}}\left(1+\frac{\mathrm{Ka}}{[\mathrm{H^+}]}\right)\left(1+\frac{[\mathrm{H^+}]}{\mathrm{Ka_2}}\right)\left(\frac{\mathrm{Ka}}{[\mathrm{H^+}]}\right)^2, \quad \text{(S19)}$$

$$\mathrm{B} = \mathrm{K_{12}}\left(1+\frac{[\mathrm{H^+}]}{\mathrm{Ka_2}}\right)(2[\mathrm{Cu(II)}]_0 - [\mathrm{ESH}]_0)\left(\frac{\mathrm{Ka}}{[\mathrm{H^+}]}\right)^2 + \mathrm{K_1Ka}\left(1+\frac{\mathrm{Ka}}{[\mathrm{H^+}]}\right)\left(\frac{1}{[\mathrm{H^+}]}+\frac{1}{\mathrm{Ka_1}}\right), \quad \text{(S20)}$$

$$\mathrm{C} = \mathrm{K_1}\left(\frac{\mathrm{Ka}}{[\mathrm{H^+}]}+\frac{1}{\mathrm{Ka_1}}\right)([\mathrm{Cu(II)}]_0 - [\mathrm{ESH}]_0) + 1 + \frac{\mathrm{Ka}}{[\mathrm{H^+}]}, \quad \text{(S21)}$$

$$D = -[ESH]_0. \tag{S22}$$

This equation was solved numerically with "roots" procedure in Matlab, and then the concentrations of other components were calculated using [ESH]:

$$[Cu(II)] = \frac{[Cu(II)]_0}{1+K_1\left(\frac{Ka}{[H^+]}+\frac{Ka}{Ka_1}\right)[ESH]+K_{12}\left(\frac{Ka}{[H^+]}\right)^2\left(1+\frac{[H^+]}{Ka_2}\right)[ESH]^2}, \tag{S23}$$

$$[C_2] = K_{12}\left(\frac{Ka}{[H^+]}\right)^2 [Cu(II)][ESH], \tag{S24}$$

$$[C_1] = K_1 \frac{Ka}{[H^+]}[Cu(II)][ESH]. \tag{S25}$$

To perform time integration of slowly changing variables we replaced differential equations (S9) – (S13) with the following explicit differential scheme:

$$\frac{C2(t+\Delta t)-C2(t)}{\Delta t} = -2k_3C2(t))^2, \tag{S26}$$

$$\frac{C3(t+\Delta t)-C3(t)}{\Delta t} = 2k_3C3(t))^2, \tag{S27}$$

$$\frac{ESSE(t+\Delta t)-ESSE(t)}{\Delta t} = k_3C2(t))^2 - k_4ESSE(t), \tag{S28}$$

$$\frac{ESH(t+\Delta t)-ESH(t)}{\Delta t} = 1.5k_4ESSE(t), \tag{S29}$$

$$\frac{EH(t+\Delta t)-EH(t)}{\Delta t} = 0.5k_4ESSE(t). \tag{S30}$$

After each time step "fast" variables were recalculated taking into account the "slow" subsystem evolution. To this end, the same cubic equation for free ESH was used with the replacement of $[ESH]_0$ and $[Cu(II)]_0$ with $[ESH]_{eq}$ and $[Cu(II)]_{eq}$ – total concentrations of ESH and Cu(II), participating in the equilibrium reactions (S1)-(S3) and determined by equations (S31) and (S32):

$$[ESH]_{eq}(t+\Delta t) = [ESH]_0 - 2[ESSE](t+\Delta t) - [C3](t+\Delta t) - [EH](t+\Delta t), \tag{S31}$$

$$[Cu(II)]_{eq}(t+\Delta t) = [Cu(II)]_0 - [C3](t+\Delta t). \tag{S32}$$

Then new equilibrium concentrations entered the next step of the differential scheme and so on. In this way, we obtained simulated time profiles for all reactants and products. Model parameters were optimized by least squares (Matlab, "fminsearch") to reproduce the experimental kinetic traces. We found that using Ka_1 and Ka_2 values above the lowest pH value (pH 5.5) used in this work resulted in poor modeling of the pH dependence of the observed ESSE formation rate. For this reason, the values of the constants Ka_1 and Ka_2 were set to 4.0, which means that at any pH value, complexes C1

and C2 are in a deprotonated state. The best fit shown in Figures 6, 8, and 10 by solid lines was obtained with the parameters: $K_a = 3.16\times10^{-11}$, $K_{a1} = K_{a2} = 1.0\times10^{-4}$, $K_{12} = 1.2\times10^{17}$, $R = K_1/K_2 = 1$, $k_4 = 1.7\times10^{-5}$ s^{-1}, the value of k_3 varied from 5.4×10^{4} $M^{-1}s^{-1}$ to 1.3×10^{5} $M^{-1}s^{-1}$.
